# Checking the rules: Comparing bodychecking, head contact, and injury risk across adolescent girls', boys’, and para ice sports in Canada

**DOI:** 10.1016/j.jsampl.2026.100158

**Published:** 2026-09-21

**Authors:** Emily E. Heming, Brooke Dennett, Ash T. Kolstad, Carolyn A. Emery, Stephen W. West

**Affiliations:** a Sport Injury Prevention Research Centre, Faculty of Kinesiology, University of Calgary, Calgary, Alberta, Canada; b O'Brien Institute for Public Health, University of Calgary, Calgary, Alberta, Canada; c Alberta Children's Hospital Research Institute, University of Calgary, Canada; d Hotchkiss Brain Institute, University of Calgary, Canada; e McCaig Institute for Bone and Joint Health, University of Calgary, Canada; f Community Health Sciences, Cumming School of Medicine, University of Calgary, Canada; g Department of Pediatrics, Cumming School of Medicine, University of Calgary, Canada; h UK Collaborating Centre on Injury and Illness Prevention in Sport, University of Bath, United Kingdom; i Centre for Health, and Injury and Illness Prevention in Sport, University of Bath, United Kingdom

**Keywords:** Ringette, Ice hockey, para ice hockey, Injury, Concussion, Video analysis

## Abstract

**Objective:**

To compare incidence rates (IR) of bodychecking (BC), head contacts (HC), and suspected injuries across Canadian adolescent ice sports (girls' ice hockey, ringette, boys’ ice hockey, and para ice hockey) via a cross-sectional video-analysis study.

**Methods:**

Video-recordings were obtained from 144 games played between 2008 and 2022 (2021–2022 for ringette, girls', and para ice hockey; 2008–2021 for boys’ ice hockey) in Canadian arenas. Video-recordings were analyzed using Dartfish video-analysis software to compare all ice sports. Univariate Poisson regression analyses (adjusted for cluster by team, offset by game-minutes) were used to estimate BC, HC, and suspected injury IRs and incidence rate ratios (IRRs) to compare rates across sports. Proportions of BCs and direct HC (HC_1_) penalized were reported.

**Results:**

Compared with non-elite non-BC boys' ice hockey, there were significantly higher rates of BC in non-BC girls' ice hockey (IRR = 2.67, 95%CI: 1.82–3.90), non-BC ringette (IRR = 5.72, 95%CI: 4.03–8.11), and BC-permitted boys' ice hockey (IRR_elite_ = 9.15, 95%CI: 6.63–12.64; IRR_non-elite_ = 5.89, 95%CI: 4.07–8.53). HC rates were significantly higher for non-BC girls' ice hockey (IRR = 2.76, 95%CI: 2.05–3.71), non-BC ringette (IRR = 4.06, 95%CI: 3.02–5.46), and BC-permitted boys’ ice hockey (IRR_elite_ = 3.18, 95%CI: 2.41–4.18; IRR_non-elite_ = 2.64, 95%CI: 1.92–3.62). Para ice hockey did not significantly differ for both BC and HC. The proportions of player-to-player HCs penalized ranged from 0 to 20% across sports.

**Conclusions:**

The high rates of BC are concerning in ice sports prohibiting BC (ringette and girls’ ice hockey). The low proportion of penalized HCs across all ice sports suggests a need for improved policy enforcement to prevent injuries and concussions.

**Key points**

• This study provides the first multi-sport comparison of bodychecking, head contact, and suspected injury and concussion events across Canadian adolescent ice sports using a consistent video-analysis protocol.
• Substantial differences in bodychecking and head contact rates were observed between sports, with ringette and boys' ice hockey showing the highest frequencies and para ice hockey the lowest.
• Penalty rates for illegal head contacts were low across all four sports, highlighting a gap between policy intent and on-ice implementation and identifying opportunities for strengthening enforcement and education to support injury prevention.

## 1 Introduction

Popular ice sports amongst Canadian adolescents include ice hockey, para ice hockey, and ringette [1,2]. Although these sports have similar rules/objectives (e.g., skating, team structure), they do have differences in rules and equipment [3,4]. Para ice hockey is a seated adaptation of stand-up ice hockey, where athletes with disabilities compete sitting in sleds fitted with a central blade and use two sticks with metal picks for propulsion [5]. Ringette is a stand-up team ice sport in which players use a straight, bladeless stick to pass, carry, and shoot a hollow rubber ring, rather than a using a bladed hockey stick and puck [3].

Participation in these team ice sports is widespread. From a 2022 parent survey of physical activity and sport participation, among the top 15 sports reported by Canadian children and youth, hockey and ringette ranked third, reported by 16% of sport participants, behind soccer and basketball [6]. Hockey Canada reported 589,012 registered athletes in the 2023-24 season, including 108,313 girls and women and 932 para ice hockey players (179 girl and woman para ice hockey players) [2]. During the 2024-25 season, Ringette Canada reported 33,657 registered athletes, with 31,941 identifying as girls or women, 1,333 as boys or men, and 240 as non-binary [1]. These numbers demonstrate that these team ice sports are among the most widely played organized sports for Canadian girls and women, highlighting the importance of gaining a better understanding of the injury and concussion risk. Although this study focuses on Canadian data, these ice sports are played internationally (ice hockey: 70 countries [7]; ringette: 11 countries [8]) under similar rules, making the findings relevant to broader injury prevention contexts.

Injury and concussion prevention is a priority in adolescent ice sports because previous surveillance and video-analysis studies have reported substantial rates of bodychecking (BC; high intensity trunk physical contact), head contacts (HCs; both direct player-to-head contacts and indirect head-to-boards/glass/ice), and suspected injuries [[9], [10], [11], [12], [13], [14]]. Although Canadian policies prohibit BC in ringette and girls' ice hockey and penalize direct HCs in all ice sports, these rules are not consistently enforced in practice [15]. A few video-analysis studies have independently examined injury mechanisms in each sport, but no prior study has directly compared these rates across all team ice sports using standardized video-analysis methods [[9], [10], [11], [12], [13], [14]]. Comparing BC, HC, suspected injuries and concussions, and penalty enforcement across these ice sports is essential for developing holistic, equitable injury prevention strategies. Therefore, the objective of this study was to compare incidence rates of BC, HC, suspected injuries and concussions, and the proportion of penalized direct HCs across Canadian adolescent boys' and girls’ ice hockey, para ice hockey, and ringette using standardized video-analysis methods.

## 2 Materials & methods

### 2.1 Study design & setting

This cross-sectional video-analysis study included 144 games from Canadian adolescent and para ice sports spanning multiple seasons (Table 1). Although footage originated from different years, all videos were analyzed using identical operational definitions and procedures [16], ensuring comparability across sports and time periods. Subsets of these data have been reported previously in single-sport analyses using similar video-analysis methods [[9], [10], [11], [12], [13], [14]].

**Table 1:** Game footage characteristics by sport, age group, and competition level.

Sport/cohort	Games (n)	Seasons	Bodychecking permitted	Participants	Age group(s)	Competition level
Boys' ice hockey	26	2015–2017	No	Boys	Under-15 & under-18	Non-elite (divisions 3–6)
	26	2014–2017	Yes	Boys	Under-15 & under-18	Non-elite (divisions 3–6)
	35	2008–2009, 2013–2014, 2020–2021	Yes	Boys	Under-15	Elite (AA)
Girls' ice hockey	8	2021–2022	No	Girls	Under-15	Elite (AA)
Ringette	39	2021–2022	No	Mixed (mainly girls)	Under-16 & under-19	Elite (AA and A)
Para ice hockey	10	2021–2022	Yes	Mixed	Not defined (adolescent and adult)	Elite (Intermediate C)

Note: In Canada, elite (AA) represents the highest competitive level within an age group, while non-elite divisions (e.g., divisions 3–6) represent community-based competitive levels below elite.

### 2.2 Video footage collection

Games were recorded in Canadian arenas using a high-definition Panasonic Full HD HC-V770 video camera mounted on a tripod. Footage was recorded at 1080p resolution and 60 frames per second. All study footage was recorded using this camera model and recording setting. Footage was research-recorded rather than broadcast footage. Cameras were located at an elevated position near centre ice whenever possible, with the next location being at the corner at ice level when the ideal position was not available (e.g., no/low stands, netting). Camera operators were trained to maintain the play in the frame, following the puck/ring carriers, display the scoreboard during stoppages, and capture referee signals to document penalties. All video-recordings were stored on a secure, access-restricted university server (SharePoint) and were not publicly accessible.

### 2.3 Outcome measures

Game footage was analyzed using Dartfish v10.0 [17], which allows for frame-by-frame tagging of player-to-player physical contacts (PCs) through a custom-built coding panel. Trained video-analysts with experience in each sport (e.g., player, coach) completed inter-rater reliability (≥90%) with a gold standard assessor in each sport on all contact metrics prior to independent coding. Player-to-player PCs were coded using validated operational definitions of trunk contact intensity, HC type, and sled contact configuration (for para ice hockey only) (Table 2). Example videos of each contact type were used alongside the definitions to support consistent interpretation and coding. Head contacts were categorized according to the source of the visible external contact to the head: player-to-head contacts (direct; HC_1_) or environment-to-head (indirect; HC_2_). Indirect inertial head-loading events without visible external contact to the head were not coded in this study.

**Table 2:** Trunk, head, sled, and other contact definitions used for video analysis in adolescent ice sports [9,10,11,12,13,14].

	Definition
Trunk physical contacts	
Level 1	Very light contact between two stationary players.
Level 2	Light contact between two players moving in the same relative direction.
Level 3	Moderate contact between two players moving in the same relative direction.
aLevel 4	Heavy contact, with one player forcefully exerting one's body into the opposing player, usually moving in the opposite direction. Minimum requirement of a body contact penalty.
aLevel 5	Excessive, deliberate contact from one player with the intention beyond impeding the progress of the opponent, moving in the opposite direction.
Head contacts (HC)	
aDirect head contact (HC_1_)	Direct contact to the head by the opposing player.
Indirect head contact (HC_2_)	Indirect contact to the head via the boards, glass, net, or ice surface.
bSled physical contacts	
aT-bone	The nose of one player's sled contacts the side of another player's sled at a 90-degree angle.
Angled	The nose of one player's sled contacts the side of another player's sled at any non-90-degree angle.
Nose-to-Nose	Player sleds contact each other head on.
Nose-to-Back	Contact player initiates contact with the front of their sled to the back of the receiving player's sled.
Other physical contacts	
Limb	Contact using one or both upper extremities, such as pushing, punching, or holding.
Stick	Contact to the body using the stick, such as slashing, hooking, or cross checking.

a Indicates contacts that should always be penalized by referees in leagues that don't allow body checking, head contacts, or t-bone sled contacts.

b Indicates contacts that were only used for para ice hockey.

No linked medical injury database was available for this cross-sport video-analysis study. Therefore, injury outcomes were classified as video-suspected injuries and suspected concussions based on observable signs. Suspected injuries were identified when one or more of the following validated criteria were observed: referee stopped play for injury, player remained on the ice for longer than 15 seconds after contact, and the player appeared to be in pain [18]. These video-based definitions have been validated previously in youth and non-elite sport settings, including studies using trained non-clinician coders [19]. Suspected concussions were defined as events meeting the suspected injury criteria plus at least one observable sign consistent with expert consensus (e.g., lying motionless, dazed, slow to get up, clutching at head) [20].

### 2.4 Data analysis

Following video coding, tagged event-level data were exported from Dartfish and merged with game-level information, including sport/cohort, team, game duration, BC policy, and competition level. Data were checked for duplicate, missing, and implausible tags before being imported into Stata for analysis. All data was analyzed using STATA version 17.1 statistical software [21]. Univariate Poisson regression analyses (adjusted for cluster by team and offset by game-minutes) were used to estimate incidence rates (IRs; expressed as the number of contacts per 100 game-minutes with 95% confidence intervals [CIs]) and incidence rate ratios (IRRs with 95% CIs) by sport and BC policy. Non-elite non-BC boys’ ice hockey served as the reference group for these comparisons. Descriptive statistics were used to summarize the proportion of penalized illegal HCs.

To assess the potential influence of temporal differences in gameplay, a sensitivity analysis was conducted excluding boys’ elite ice hockey games from the 2008-09 season. IRs and IRRs were not significantly different when compared to the main analyses, and the overall pattern of results did not change, therefore, all years were retained in the final analyses.

### 2.5 Data management and ethics

All coded data and video files were stored on secure university servers. No player-identifiable information was collected, and analyses were limited to observable on-ice behaviour. Ethical approval for this study was obtained from the University of Calgary Conjoint Health Research Ethics Board (CHREB) (Ethics ID: REB21-0968).

## 3 Results

Numbers of games, game-minutes, and outcome counts (i.e., number of bodychecks, head contacts, suspected injuries, etc.) are provided in Table 3.

**Table 3:** Total game-minutes, bodychecks, head contacts, and suspected injuries across adolescent ice sports.

Sport	Games (n)	Game-minutes	Bodychecks (n)	Total head contacts (n)	Direct head contacts (n)	Indirect head contacts (n)	All suspected injuries (n)	Suspected non-concussion injuries (n)	Suspected concussion injuries (n)
**Boys' ice hockey** (non elite non-BC)	26	1170	71	110	101	9	7	3	4
**Girls' ice hockey** (elite non-BC)	8	482	78	125	87	38	6	3	3
**Ringette** (elite non-BC)	39	1629	565	622	447	175	50	26	24

**Para ice hockey** (elite BC)	10	360	33	48	43	5	2	1	1
**Boys' ice hockey** (non elite BC)	26	1170	418	290	290	0	13	8	5
**Boys' ice hockey** (elite BC)	35	1899	1055	567	378	189	26	15	11

Note: All suspected injuries includes both suspected non-concussions and suspected concussions.

### 3.1 Bodychecking

BC incidence differed significantly across sports (Table 4). Compared with boys' non-BC ice hockey, rates were significantly higher in girls' ice hockey, ringette and BC-permitted boys' ice hockey. Both elite and non-elite BC-permitted boys' ice hockey demonstrated the highest rates, followed by ringette and girls’ ice hockey, despite BC being prohibited in the latter two sports. Para ice hockey did not statistically differ from the reference group.

**Table 4:** Incidence rates (IRs) per 100 game-minutes and incidence rate ratios (IRRs) for bodychecking (BC) across adolescent ice sports. Boys’ non-bodychecking (non-BC) ice hockey served as the reference group.

Sport	Bodychecking (BC)	
	IR/100 game-minutes (95% CI)	IRR (95% CI)
**Boys' ice hockey** (Non elite non-BC)	6.07 (4.51–8.16)	(Reference)
**Girls' ice hockey** (Elite non-BC)	16.18 (12.46–21.00)	2.67 (1.82–3.90)∗
**Ringette** (Elite non-BC)	34.68 (28.58–42.09)	5.72 (4.03–8.11)∗

**Para ice hockey** (Elite BC)	9.17 (5.13–16.39)	1.51 (0.81–2.82)
**Boys' ice hockey** (Non elite BC)	35.73 (28.34–45.03)	5.89 (4.07–8.53)∗
**Boys' ice hockey** (Elite BC)	55.56 (48.40–63.76)	9.15 (6.63–12.64)∗

Notes: ∗Indicates a statistically significant difference (95% CI does not include 1) compared with boys' non-BC ice hockey.

### 3.2 Head contacts

HC incidence also differed significantly across sports (Table 5). Compared with boys' non-BC ice hockey, girls' ice hockey, ringette, and both BC-permitted boys' ice hockey divisions showed significantly higher total HC rates. Para ice hockey did not differ statistically from the reference group. When stratified by HC type, all sports except para ice hockey demonstrated significantly higher HC_1_ rates than the reference group. HC_2_ rates were significantly higher in girls' ice hockey, ringette, and elite BC-permitted boys’ ice hockey.

**Table 5:** Incidence rates (IRs) per 100 game-minutes and incidence rate ratios (IRRs) for total, direct (HC_1_), and indirect (HC_2_) head contacts (HCs) across adolescent ice sports. Boys’ non-bodychecking (non-BC) ice hockey served as the reference group.

Sport	Total head contact (HC)		Direct head contact (HC_1_)		Indirect head contact (HC_2_)	
	IR/100 game-minutes (95% CI)	IRR (95% CI)	IR/100 game-minutes (95% CI)	IRR (95% CI)	IR/100 game-minutes (95% CI)	IRR (95% CI)
**Boys' ice hockey** (Non elite non-BC)	9.40 (7.25–12.19)	(Reference)	8.63 (6.49–11.49)	(Reference)	0.77	(Reference)
**Girls' ice hockey** (Elite non-BC)	25.92 (22.05–30.48)	2.76 (2.05–3.71)∗	18.04 (15.11–21.54)	2.09 (1.51–2.90)∗	7.88 (6.05–10.27)	10.24 (4.37–24.00)∗
**Ringette** (Elite non-BC)	38.18 (32.91–44.30)	4.06 (3.02–5.46)∗	27.44 (23.44–32.13)	3.18 (2.30–4.39)∗	10.74 (8.91–12.96)	13.96 (6.06–32.19)∗

**Para ice hockey** (Elite BC)	13.33 (8.66–20.02)	1.42 (0.70–2.88)	11.94 (6.69–18.83)	1.38 (0.67–2.86)	1.39 (0.58–3.34)	1.81 (0.56–5.79)
**Boys' ice hockey** (Non elite BC)	24.79 (20.50–29.97)	2.64 (1.92–3.62)∗	24.79 (20.50–29.97)	2.87 (2.05–4.02)∗	- (no events observed)	- (no events observed)
**Boys' ice hockey** (Elite BC)	29.86 (26.93–33.10)	3.18 (2.41–4.18)∗	19.91 (17.53–22.61)	2.31 (1.69–3.14)∗	9.95 (8.37–11.84)	12.94 (5.63–29.73)∗

Notes: ∗Indicates a statistically significant difference compared with boys' non-BC ice hockey.

### 3.3 Suspected injuries and concussions

Suspected injury outcomes varied by sport (Table 6). Compared with boys’ non-BC ice hockey, ringette showed significantly higher rates for total suspected injuries, as well as both concussion and non-concussion injuries. No other sports were statistically different from the reference group for all injuries or when broken down into concussion and non-concussion injuries.

**Table 6:** Incidence rates (IRs) per 100 game-minutes and incidence rate ratios (IRRs) for total suspected injuries, concussion injuries, and non-concussion injuries across adolescent ice sports. Boys’ non-bodychecking (non-BC) ice hockey served as the reference group.

Sport	Total injuries		Non-concussion injuries		Concussion injuries	
	IR/100 game-minutes (95% CI)	IRR (95% CI)	IR/100 game-minutes (95% CI)	IRR (95% CI)	IR/100 game-minutes (95% CI)	IRR (95% CI)
**Boys' ice hockey** (Non elite non-BC)	0.60 (0.28–1.28)	(Reference)	0.26 (0.09–0.76)	(Reference)	0.34 (0.14–0.86)	(Reference)
**Girls' ice hockey** (Elite non-BC)	1.24 (0.65–2.39)	2.08 (0.79–5.47)	0.62 (0.24–1.62)	2.43 (0.60–9.81)	0.62 (0.24–1.61)	1.82 (0.51–6.49)
**Ringette** (Elite non-BC)	3.07 (2.15–4.38)	5.13 (2.24–11.74)∗	1.60 (0.93–2.73)	6.22 (1.89–20.52)∗	1.47 (0.90–2.41)	4.31 (1.54–12.05)∗

**Para ice hockey** (Elite BC)	0.56 (0.15–2.05)	0.93 (0.22–3.97)	0.28 (0.04–1.97)	1.08 (0.13–9.30)	0.28 (0.04–1.97)	0.81 (0.10–6.46)
**Boys' ice hockey** (Non elite BC)	1.11 (0.65–1.91)	1.86 (0.74–4.66)	0.68 (0.34–1.36)	2.67 (0.75–9.43)	0.43 (0.19–0.95)	1.25 (0.38–4.16)
**Boys' ice hockey** (Elite BC)	1.37 (0.92–2.04)	2.29 (0.98–5.34)	0.79 (0.46–1.35)	3.08 (0.93–10.15)	0.58 (0.32–1.06)	1.69 (0.57–5.01)

Notes: ∗Indicates a statistically significant difference compared with boys' non-BC ice hockey.

### 3.4 Penalty enforcement

Across all sports and despite illegality, direct head contacts (HC_1_) were rarely penalized, with enforcement ranging from 0% to 20% (Table 7). The highest proportion of illegal HC_1_s penalized were observed in boys' ice hockey, with a lower proportion in girls’ ice hockey and ringette, and no penalization of illegal HC_1_s observed in para ice hockey.

**Table 7:** Number and proportion of illegal direct head contacts (HC_1_) penalized across adolescent ice sports.

Sport	Direct head contacts, n	Direct head contacts penalized, n	Penalized, %	95% CI
**Boys' ice hockey** (non elite non-BC)	101	20	20	13 to 29
**Girls' ice hockey** (elite non-BC)	87	5	6	2 to 13
**Ringette** (elite non-BC)	447	31	7	5 to 10
**Para ice hockey** (elite BC)	43	0	0	0 to 8
**Boys' ice hockey** (non elite BC)	290	32	11	8 to 15
**Boys' ice hockey** (elite BC)	378	60	16	12 to 20

Note: BC = bodychecking; CI = confidence interval.

## 4 Discussion

This study provides the first direct comparison of BC, HC, and suspected injury rates across adolescent boys' and girls' ice hockey, para ice hockey, and ringette using standardized video-analysis methods. In ringette, girls' ice hockey, and some boys' ice hockey leagues, bodychecking is prohibited by sport rules, meaning intentional high-intensity trunk contact should be penalized rather than treated as permitted gameplay. Rates of BC and HC differed across sport cohorts. Ringette and girls' ice hockey demonstrated higher BC and HC rates than boys’ non-BC ice hockey, despite BC being prohibited in these sports, and ringette demonstrated the highest suspected injury and concussion rates. These findings suggest discrepancies between written sport policies and on-ice behaviours and highlight opportunities to strengthen enforcement, education, and sport-specific prevention strategies in adolescent ice sports.

### 4.1 Bodychecking and head contacts

The significantly higher BC and HC rates observed in ringette and girls' ice hockey suggest that rule infractions are occurring frequently without corresponding officiating response. The higher BC and HC rates observed in girls' ice hockey compared with boys’ non-BC ice hockey may reflect several interacting contextual factors, including differences in contact experience, instruction in anticipating or absorbing contact, motor skills, officiating expectations, and broader sport culture. These explanations remain speculative, as these factors were not measured directly in this study, but they may be particularly relevant in non-BC settings, where athletes may still experience high-intensity contact but may receive less contact-specific training than athletes in BC-permitted leagues. This is consistent with earlier single-sport video-analysis studies showing that a substantial proportion of BCs and HCs occur unpenalized in female adolescent leagues [[9], [10], [11], [12], [13], [14]]. These findings reinforce the need for standardized officiating education emphasizing the identification and penalization of illegal contact across all levels of play. Although officiating education and safety initiatives exist within Canadian ice sports, consistency and uptake vary by organization and level of play [15]. The absence of penalization for illegal direct HCs in para ice hockey is of particular concern and suggests that officiating enforcement may be even less consistent within this population.

### 4.2 Suspected injuries and concussions

Ringette demonstrated both high rates of bodychecking and the highest suspected injury and concussion rates, aligning with previous research [[12], [13], [14]]. This pattern, together with previous youth ice hockey evidence linking bodychecking policy and physical contact to injury risk, supports continued attention to illegal high-intensity contact as a potential prevention target. Possible explanations include sex-based differences in neck strength [22], neuromuscular control [23], and hormonal factors [24], alongside contextual elements such as rule differences, faster game transitions, and less protective equipment [3,4]. Given the high rates of prohibited bodychecking in non-bodychecking sport cohorts and the low penalization of direct head contacts across ice sports, the first intervention to explore should be stronger implementation of existing contact-related rules. This could include education and structural support for officials, such as preseason video-based calibration, clearer rule-interpretation guidance, and feedback processes to support consistent identification and penalization of illegal contact [15]. Aligned education for coaches, players, and parents may also be needed to reinforce that penalty enforcement is an injury prevention strategy rather than a disruption to game flow. Other complementary prevention approaches may include contact-specific skill training to help athletes anticipate, avoid, and safely respond to contact; neuromuscular training warm-ups to address modifiable movement and strength-related injury risk factors; and equipment evaluation to determine whether protective equipment influences contact behaviour or injury risk [25]. However, these strategies require sport-specific evaluation before conclusions can be drawn about their effectiveness in reducing suspected injuries or concussions in these cohorts.

These findings align with recent consensus work emphasizing that injury prevention strategies developed in boys' and men's sport should not be assumed to translate directly to female/woman/girl athletes or para-athletes without considering biological, biomechanical, equipment, policy, and social-context differences [[25], [26], [27]]. In the present study, differences in bodychecking, head contact, suspected injury, and suspected concussion rates across sport cohorts support the need for prevention strategies that are tailored to the rules, contact context, and participants in each sport.

Evidence from other youth team sports supports the importance of contact mechanism (observable characteristics of the contact event, including body region contacted, whether the head was contacted by another player or the playing environment, contact intensity, and whether the event was penalized), rather than overall contact frequency alone, in shaping concussion risk [28]. Rugby video-analysis research demonstrates that concussion risk is concentrated within contact characteristics, including direct head involvement, higher tackle height, and high-speed or forceful player interactions [28]. The legality of contact depends on the sport, league, body region contacted, contact type, and game context. Therefore, the prevention issue is not simply the presence of contact, but whether contacts that are prohibited or high-risk under existing rules are consistently identified and penalized. This is consistent with the present findings, where player-to-head contacts occurred across sport cohorts but were infrequently penalized.

### 4.3 Penalty enforcement

Across the included ice sports, a maximum of 20% of HC_1_s results in a penalty call, revealing a substantial gap between policy intent and on-ice practice. However, low penalization should be interpreted considering the realities of officiating in real time. Unlike video analysts, referees must make rapid decision during dynamic play, often without a clear view of every player and without the ability to pause, rewind, or review the event from multiple angles. These perceptual and time constraints occur alongside social pressures from players, coaches, and parents, which may further influence consistency in identifying and penalizing illegal contacts [15]. Together, these demands may contribute to under-calling even when officials understand the rules, suggesting that improved enforcement likely requires structural support in addition to education.

Embedding this support within sport organizations, through pre-season education and integration into coaching and officiating certification, could foster safer playing environments and normalize consistent enforcement of BC and HC policies. Complementary structural strategies may also be needed, such as reducing barriers to implementation, providing officials with more support or resources, or adjusting officiating structures to lessen the physical and perceptual load placed on individual referees. These broader changes acknowledge that consistent rule enforcement depends not only on individual capability, but also on opportunity and motivation shaped by the wider sport environment [29].

## 5 Limitations

There are several limitations to consider in this cross-sport video-analysis study, reflecting inherent challenges of video-based injury surveillance. First, although standardized coding definitions and training were used, video quality and camera positioning varied across games and seasons. Limited visibility of off-puck/ring or off-camera events may have led to an underestimation of contacts and suspected injuries. As well, identification of suspected injuries and concussions is based on observable signs rather than medical confirmation. This may underestimate true injury and concussion incidence, particularly for events with delayed symptom onset or limited visible signs. However, because these video-based limitations were likely present across all included ice sports, these limitations are expected to have affected overall event counts rather than explaining the observed between-sport differences. Second, exposure duration differed by sport (e.g., 40-min ringette vs 60-min ice hockey games), but rates were normalized per 100 game-minutes to minimize this bias. Third, the para ice hockey data were derived from a single tournament with fewer games. This may result in less precise estimates and limiting generalizability to broader para ice hockey settings. Fourth, although footage spanned multiple seasons, sensitivity analyses excluding the earliest cohort showed no meaningful impact on outcomes. However, residual temporal differences may remain. Finally, differences in competitive level (elite vs non-elite), age group, and participant characteristics across cohorts may have influenced gameplay intensity and contact patterns. These differences reflect the natural structure of adolescent sport participation in Canada, and the divisions analyzed represented the most comparable competitive levels across the included ice sports, but should be considered when interpreting cross-sport comparisons [3,5,30].

## 6 Perspective

This study provides novel cross-sport video-analysis evidence highlighting discrepancies between injury prevention policy and on-ice practice across adolescent ice sports. The presence of BC and HC in sports prohibiting these actions underscores the need for enhanced enforcement, education, and culture change around contact behaviour. Referees, coaches, players, and parents collectively influence game norms and can either reinforce or hinder rule compliance. These findings extend previous single-sport analyses and complement recent discussions positioning referees as critical facilitators of injury prevention policy implementation. Future work should evaluate interventions targeting officiating consistency and interest holder education to strengthen policy adherence and reduce injury and concussion risk in adolescent team ice sports.

## 7 Conclusion

This multiple ice sport video-analysis study highlights that despite prohibiting BC, adolescent ringette and girls' ice hockey demonstrated high rates of BCs compared to boys’ ice hockey that also prohibits BC. Ringette showed the highest suspected injury and concussion rates, and across all sports, enforcement of illegal HCs penalties was low. Collectively, these findings emphasize the urgent need for improved rule enforcement, referee education, and the implementation of tailored prevention strategies addressing sex-, gender-, and sport-specific risks in adolescent ice sports.

## Declaration of competing interest

EEH and BD have no conflicts of interest to disclose. ATK holds doctoral funding through the UCalgary Eyes High Doctoral Recruitment Scholarship, Canadian Institutes of Health Research Frederick Banting and Charles Best Doctoral Scholarship, and the Izaak Walton Killam Pre-Doctoral Award. He is an external scientific member of two Hockey Canada concussion prevention working groups (unpaid). CAE holds a Canada Research Chair (Tier 1) in Concussion and research funding from Canadian Institutes of Health Research, Canada Foundation for Innovation, IOC, World Rugby and National Football League Play Smart Play Safe Program. SWW holds research funding from World Rugby.

## References

[1] Ringette Canada annual report 2024-2025.pdf. https://www.ringette.ca/wp-content/uploads/2025/09/Annual-Report-2024-25-FINAL-EN.pdf.

[2] Hockey Canada annual report 2023-2024. https://cdn.hockeycanada.ca/hockey-canada/Corporate/About/Downloads/2023-24-hockey-canada-annual-report-e.pdf.

[3] Official Rules and Case Book 2025-2027 Ringette Canada. https://www.ringette.ca/about-us/rule-book/.

[4] Hockey Canada playing rules 2024-2026. https://cdn.hockeycanada.ca/hockey-canada/Hockey-Programs/Officiating/Downloads/rulebook_casebook_e.pdf.

[5] Para Hockey Development. https://www.hockeycanada.ca/en-ca/hockey-programs/sledge.

[6] Sport Participation among children and youth Canadian fitness and lifestyle Research Institute. https://cflri.ca/publication/sport-participation-among-children-and-youth/.

[7] 2024 IIHF Annual Survey of players. vol. 3. Accessed May 26, 2026. https://blob.iihf.com/iihf-media/iihfmvc/media/2025/survey/2024_iihf_annual_sop_iihf_com.pdf.

[8] Ringette History National ringette school. https://nationalringetteschool.com/ringette/facts.

[9] Williamson R.A., Cairo A.L., Heming E.E., Kolstad A.T., Hagel B.E., Emery C.A. (2023). Physical contact and suspected injury rates in female versus Male youth ice hockey: a video-analysis study. Clin J Sport Med.

[10] Dennett B. (2024). ME09 – moderated E-Poster Session 02 – station 3, Station 3, March 1, 2024, 12:30 – 13:30.

[11] Kolstad A.T., Chin M., Martinez G., Janzen L., Krolikowski M., Goulet C. (2022). Evaluating the effect of Policy prohibiting body checking on physical contacts in U15 and U18 youth ice hockey leagues. Clin J Sport Med.

[12] Heming E.E., Kolstad A.T., West S.W., Williamson R.A., Sobry A.J., Galarneau J.M. (2026). Blow the whistle: a cross-sectional video-analysis study examining bodychecking, suspected injuries and penalisation of illegal contacts in youth ringette. BMJ Open Sport Exerc Med.

[13] Heming E.E., Sobry A.J., Cairo A.L., Williamson R.A., Kolstad A.T., West S.W. (2023). Higher rates of head contacts, body checking, and suspected injuries in ringette than female ice hockey: time to ring in opportunities for prevention. Clin J Sport Med.

[14] Heming E.E., Kolstad A.T., West S.W., Williamson R.A., Sobry A.J., Cairo A.L. (2024). Time to ring in the body checking, head contact, and suspected injury rates in youth ringette: a video-analysis Study in youth ringette and female ice hockey. Clin J Sport Med.

[15] West S.W., Heming E.E., Klassen C., Lewis K., Emery C.A. (2025). Whistleblower: the front line and critical facilitator for injury prevention policy?. Inj Prev.

[16] Malenfant S., Goulet C., Nadeau L., Hamel D., Emery C.A. (2012). The incidence of behaviours associated with body checking among youth ice hockey players. J Sci Med Sport.

[17] Dartfish V 10.0. Dartfish Video analysis software.

[18] Andersen T.E. (2003). Football incident analysis: a new video based method to describe injury mechanisms in professional football. Br J Sports Med.

[19] West S.W., Shill I.J., Sutter B., George J., Ainsworth N., Wiley J.P. (2022). Caught on camera: a video assessment of suspected concussion and other injury events in women's rugby union. J Sci Med Sport.

[20] Davis G.A., Makdissi M., Bloomfield P., Clifton P., Echemendia R.J., Falvey É.C. (2019). International consensus definitions of video signs of concussion in professional sports. Br J Sports Med.

[21] StataCorp (2021). Stata Statistical Software: Release.

[22] Williams E.M.P., Petrie F.J., Pennington T.N., Powell D.R.L., Arora H., Mackintosh K.A. (2022). Sex differences in neck strength and head impact kinematics in university rugby union players. Eur J Sport Sci.

[23] Landry S.C., McKean K.A., Hubley-Kozey C.L., Stanish W.D., Deluzio K.J. (2009). Gender differences exist in neuromuscular control patterns during the pre-contact and early stance phase of an unanticipated side-cut and cross-cut maneuver in 15–18 years old adolescent soccer players. J Electromyogr Kinesiol.

[24] La Fountaine M.F., Hill-Lombardi V., Hohn A.N., Leahy C.L., Testa A.J. (2019). Preliminary evidence for a window of increased vulnerability to sustain a concussion in females: a brief report. Front Neurol.

[25] Crossley K.M., Whittaker J.L., Patterson B., Shill I.J., Heming E.E., Bullock G.S. (2025). Female, woman and/or girl Athlete Injury pRevention (FAIR) practical recommendations: International Olympic Committee (IOC) consensus meeting held in Lausanne, Switzerland, 2025. Br J Sports Med.

[26] Heming E.E., West S.W., Patterson B., Bullock G.S., McHugh T.L., Shill I.J. (2025). Raising girls in sport: unique considerations for injury prevention. Br J Sports Med.

[27] Grimes A.C., Harrington (Quinn) N., Blauwet C., Ahmed O.H., Gavel-Pinos E.H., Mitchell K.M. (2025). Towards an evidence-informed future in injury prevention: a call to action for female, women and girls in para sport. Br J Sports Med.

[28] Shill I.J., West S.W., Galarneau J.M., Omokuale J., Hendricks S., Pike I. (2025). Tackle characteristics and suspected concussion: recommendations to improve high school girls' rugby tackle safety. BMJ Open Sport Exerc Med.

[29] Michie S., Van Stralen M.M., West R. (2011). The behaviour change wheel: a new method for characterising and designing behaviour change interventions. Implement Sci.

[30] Hockey Canada Age Divisions | Age range info for minor hockey. https://www.hockeycanada.ca/en-ca/hockey-programs/players/essentials/age-divisions.

